# Effects of Nerve Growth Factor-β From Bull Seminal Plasma on Steroidogenesis and Angiogenic Markers of the Bovine Pre-ovulatory Follicle Wall Cell Culture

**DOI:** 10.3389/fvets.2021.786480

**Published:** 2022-01-17

**Authors:** Jamie L. Stewart, Liying Gao, Jodi A. Flaws, Vitor R. G. Mercadante, Nicholas W. Dias, Igor F. Canisso, Fabio S. Lima

**Affiliations:** ^1^Department of Large Animal Clinical Sciences, Virginia-Maryland College of Veterinary Medicine, Virginia Polytechnic Institute and State University, Blacksburg, VA, United States; ^2^Department of Comparative Biosciences, College of Veterinary Medicine, University of Illinois, Urbana, IL, United States; ^3^Department of Veterinary Clinical Medicine, College of Veterinary Medicine, University of Illinois, Urbana, IL, United States; ^4^Department of Animal and Poultry Sciences, Virginia Polytechnic Institute and State University, Blacksburg, VA, United States; ^5^Department of Population Health and Reproduction, School of Veterinary Medicine, University of California, Davis, Davis, CA, United States

**Keywords:** angiogenesis, NGF, granulosa cells, theca cells, ruminants

## Abstract

Nerve growth factor-β (NGF) is critical for ovulation in the mammalian ovary and is luteotrophic when administered systemically to camelids and cattle. This study aimed to assess the direct effects of purified bovine NGF on steroidogenesis and angiogenic markers in the bovine pre-ovulatory follicle. Holstein heifers (*n* = 2) were synchronized with a standard protocol, and heifers with a preovulatory follicle (≥ 12 mm) had the ovary containing the dominant follicle removed via colpotomy. Pre-ovulatory follicles were dissected into 24 pieces containing theca and granulosa cells that were randomly allocated into culture media supplemented with either purified bovine NGF (100 ng/mL) or untreated (control) for 72 h. The supernatant media was harvested for quantification of progesterone, testosterone, and estradiol concentrations, whereas explants were subjected to mRNA analyses to assess expression of steroidogenic and angiogenic markers. Treatment of follicle wall pieces with NGF upregulated gene expression of steroidogenic enzyme *HDS17B* (*P* = 0.04) and increased testosterone production (*P* < 0.01). However, NGF treatment did not alter production of progesterone (*P* = 0.81) or estradiol (*P* = 0.14). Consistently, gene expression of steroidogenic enzymes responsible for producing these hormones (*STAR, CYP11A1, HSD3B, CYP17A1, CYP19A1*) were unaffected by NGF treatment (*P* ≥ 0.31). Treatment with NGF downregulated gene expression of the angiogenic enzyme *FGF2* (*P* = 0.02) but did not alter *PGES* (*P* = 0.63), *VEGFA* (*P* = 0.44), and *ESR1* (*P* = 0.77). Collectively, these results demonstrate that NGF from seminal plasma may interact directly on the theca and granulosa cells of the bovine pre-ovulatory follicle to stimulate testosterone production, which may be secondary to theca cell proliferation. Additionally, decreased *FGF2* expression in NGF-treated follicle wall cells suggests hastened onset of follicle wall cellular remodeling that occurs during early luteal development.

## Introduction

Nerve growth factor-β (NGF) is a member of the neurotrophin family that has a critical role in mammalian follicle development and ovulation (1, 2). Expression of NGF has been localized to ovarian granulosa cells prior to the formation of the first primordial follicles in neonatal mice (2). Though present during late fetal development, expression of NGF and its receptor, TrkA, in the ovary decreases postnatally between 24 and 48 h after birth and remains low until puberty in rats (1). At the time of the first pre-ovulatory luteinizing hormone (LH) surge, transient activation of NGF/TrkA occurs, and contributes to follicular cytodifferentiation preceding the first ovulation (1). In bovine theca cells, NGF induced prostaglandin E_2_ (PGE) synthesis (3), which facilitates follicular rupture at ovulation (4). The presence of both LH and follicle-stimulating hormone (FSH) *in vitro* were necessary to stimulate NGF secretion from medium to large follicles in ewes, suggesting a synergistic role with gonadotropins during the pre-ovulatory cascade (5).

Though the ability of the ovary to produce NGF in spontaneously ovulating species is well-documented, few studies have evaluated whether the introduction of NGF from seminal plasma at time of breeding may have a role within the bovine hypothalamic-pituitary-ovarian axis (6, 7). In camelids, intrauterine absorption of seminal plasma NGF into systemic circulation occurs within 15 min of copulation, after which it stimulates the preovulatory LH peak from the anterior pituitary gland and exerts a dose-dependent luteotropic effect on the developing corpus luteum (CL) (8). Studies have shown that NGF retains its luteotropic properties when administered systemically to cattle (7). While the luteotropic effect in camelids is attributed to prolonged LH secretion from the anterior pituitary, in cattle, there is evidence that NGF may act directly on the ovary (3).

Similar to camelids, bull seminal plasma contains NGF, which is concentrated into the sperm-rich fraction of the ejaculate, facilitating its transit into the cow reproductive tract (9). The bovine uterus maintains a local countercurrent exchange between the uterine venous drainage and the ovarian artery that allows for direct transport of prostaglandin F_2α_ (PGF_2α_) from the uterus to the ovary during luteolysis (10, 11). This anatomical mechanism could also provide a potential route for NGF to travel to and interact directly with the ovary (12). A previous study demonstrated that treating bovine theca cells with recombinant NGF *in vitro* resulted in increased androstenedione and progesterone release, PGE production, and theca cell proliferation when compared to hCG-treated controls (3). However, the signaling cascade within the ovary involves a complex interaction between the theca and granulosa cells, which has yet to be elucidated. This study aimed to assess the direct effects of NGF, purified from bull seminal plasma, on steroidogenesis and angiogenic markers in theca and granulosa cells of the bovine pre-ovulatory follicle. We hypothesized that NGF administration would stimulate steroidogenesis and angiogenic markers in thecal and granulosa cells from the bovine pre-ovulatory follicle.

## Materials and Methods

### Animal Care and Use

The present study was conducted in 2018 at the College of Veterinary Medicine, University of Illinois Urbana-Champaign. The Institutional Animal Care approved all the animal procedures and Use Committees of the University of Illinois at Urbana-Champaign, USA (Protocol #18223).

### Heifer Synchronization and Ovariectomy

A cohort of three cyclic Holstein heifers (presence of corpus luteum > 16 mm and pre-ovualtory follicle > 12 mm) of ~2 years of age had their estrous cycles synchronized. The heifers received an intravaginal progesterone-releasing device (1.38 g progesterone; Eazi-Breed CIDR, Zoetis, Parsippany-Troy Hills, NJ, USA) and an injection of GnRH agonist (100 μg Factrel^®^, Zoetis, Parsippany-Troy Hills, NJ, USA) intramuscularly (day −8). The intravaginal device was removed 5 days later (day −3), and heifers were given PGF_2α_ analog (25 mg dinoprost tromethamine, Lutalyse, Zoetis, Parsippany-Troy Hills, NJ, USA) intramuscularly at the time of the intravaginal device removal and again 24 h later (day −2). Heifers were examined daily by transrectal ultrasonography to assess response to the synchronization program. At day 0 (48 h after the second PGF_2α_ injection), heifers that had undergone complete luteolysis and dominant follicle selection, as determined by the absence of a CL and the presence of a >12 mm follicle, were subjected to ovariectomy. Ovariectomy was performed via colpotomy in the standing position under caudal epidural anesthesia with 5 mL of 2% (w/v) lidocaine HCl (VetOne^®^, Boise, Idaho, USA). Only the ovary containing a dominant, pre-ovulatory follicle was removed. One heifer was excluded from the study after being unable to remove the ovary containing the dominant follicle. Heifers were treated pre-operatively with ceftiofur crystalline free acid (6.6 mg/kg; Excede^®^, Zoetis, Parsippany-Troy Hills, NJ, USA) injected subcutaneously in the base of the ear to prevent infection. Flunixin meglumine (2.2 mg/kg; Norbrook^®^ Inc., Overland Park, KS, USA) was administered intravenously daily for 2 days to control inflammation and pain. Ovaries were placed in ice-cold phosphate-buffered saline solution containing 2% antibiotic-antimycotic mixture (25 μg/mL amphotericin B, 10,000 units/mL penicillin, 10,000 μg/mL streptomycin; Gibco, Gaithersburg, MD, USA) for transport to the laboratory.

### Isolation of Follicular Wall Cells and Treatment Allocation

The pre-ovulatory follicle was identified and dissected away from each ovary for use in the follicle wall cell culture system. Follicular fluid was aspirated to facilitate further dissection and frozen at −80°C. The follicles were dissected into quarters, and the theca interna with adherent granulosa cells was peeled from the theca externa and surrounding stromal wall cells. The remaining follicle wall preparations (theca interna and granulosa cells) were cut into 26 pieces (average weight: 5.3 ± 0.7 mg), 24 of which were transferred to a costar 24-well-plate (1 piece/well; Cambridge, MA, USA) for follicle wall culture, as previously described (13, 14). The remainder of the follicle wall was flash-frozen in liquid nitrogen and maintained at −80°C until mRNA extraction.

The dissected 24 follicle wall pieces containing theca and granulosa cells were randomly allocated to receive a culture medium that was either supplemented with purified bovine NGF (100 ng/mL, *n* = 2 heifers; 6 follicle wall piece replicates from each) or left untreated (control, *n* = 2 heifers; 6 follicle wall piece replicates from each). The single plate was incubated at 37°C in a humidified incubator gassed with 5% CO_2_:95% air for 72 h. The NGF was purified from bovine seminal plasma, as described previously (7). The follicle wall pieces were cultured in 0.5 mL of medium consisting of Eagle's MEM (Invitrogen, Carlsbad, CA, USA) supplemented with 1% L-glutamine (Gibco, Gaithersburg, MD, USA), 1% non-essential amino acids (Sigma-Aldrich, St. Louis, MO, USA), 1% penicillin-streptomycin (Sigma-Aldrich), 1% ITS (10 ng/ml insulin, 5.5 ng/ml transferrin, 5.5 ng/ml selenium, Sigma-Aldrich), 10% fetal bovine serum (FBS, Atlanta Biologicals, Lawrenceville, GA), 40 ng/mL cortisol (Sigma-Aldrich), 4 ng/mL human recombinant LH (Dr. A. F. Parlow, National Hormone and Peptide Program, Harbor- UCLA Medical Center, Torrance, CA, USA), and 4 ng/mL human recombinant FSH (Dr. A. F. Parlow, National Hormone and Peptide Program).

### Hormone Assays

At each experimental time point during culture (3, 6, 12, 24, 48, and 72 h), aliquots (0.5 mL) of culture medium were collected and preserved at −20°C for subsequent steroid assays. A fresh 0.5 mL aliquot of media, containing NGF treatment or control, was added to each well at each time point except 72 h when the experiment was completed. Progesterone, testosterone, and estradiol-17β concentrations at each time point in the culture media were assessed using immunoassays (Immulite 2000 XPi platform; Siemens Medical Solutions, Malvern, PA, USA, Inc.). Total hormone production for each well was calculated by multiplying the measured concentration by the volume of media (0.5 mL) and then dividing by follicle wall cells weight (mg). Intra-assay coefficient of variations were 4.0% (testosterone), 2.4% (progesterone), and 3.1% (estradiol-17β). Inter-assay coefficient of variations were 12% (testosterone), 19% (progesterone), and 15% (estradiol-17β). The progesterone assay had a detection range of 0.2–40 ng/mL and a sensitivity of 0.1 ng/mL. The testosterone assay had a detection range of 20–1,600 ng/mL and a sensitivity of 15 ng/dL. The estradiol-17β assay had a detection range of 20–2,000 pg/mL and a sensitivity of 15 pg/mL.

### Quantitative Real-Time PCR Analyses

At the completion of the 72-h culture period, follicle wall pieces were weighed and flash-frozen and kept at −80°C until RNA extraction. Follicular wall cells mRNA expression was determined for LH/choriogonadotropin receptor (*LHCGR*), FSH receptor (*FSHR*), PGE synthase (*PGES*), vascular endothelial growth factor A isoform 121 (*VEGFA121*), fibroblast growth factor 2 (*FGF2*), estrogen receptor 1 (*ESR1*), steroidogenic acute regulatory protein (*STAR*), cytochrome P450 family 11 subfamily A member 1 (*CYP11A1*), cytochrome P450 family 17 subfamily A member 1 (*CYP17A1*), cytochrome P450 family 19 subfamily A member 1 (*CYP19A1*), hydroxyl-delta-5-steroid dehydrogenase 3-beta (*HSD3B*), and hydroxysteroid 17-beta dehydrogenase (*HSD17B*). Primers were designed for the constitutively expressed mRNAs, glyceraldehyde-3-phosphate dehydrogenase (*GAPDH*), ribosomal protein L 15 (*RPL15*), and ribosomal protein L 19 (*RPL19*), with the expression value of each gene normalized to the mean values of these genes (Table 1). Relative expression values were obtained by determining the PCR amplification efficiency (E = 2) to the power of the delta-delta threshold cycle (ΔΔCt) obtained from the ΔCt least square mean differences of pairwise comparisons between initial and cultured follicle wall cells (15).

**Table 1:** List of genes and primers used for quantitative real-time PCR.

**Target genes and abbreviations**	**NCBI Sequence**	**Primer**	**Primer sequence**
Glyceraldehyde-3-phosphate dehydrogenase (*GAPDH*)	NM_001034034	Forward Reverse	5′-GGCGCCAAGAGGGTCAT-3′ 5′-ACGCCCATCACAAACATGG-3
Ribosomal protein L 15 (*RPL15*)	AY786141	Forward Reverse	5′-TGGAGAGTATTGCGCCTTCTC-3′ 5′-CACAAGTTCCACCACACTATTGG-3′
Ribosomal protein L 19 (*RPL19*)	NM_001040515	Forward Reverse	5′-CAGACGATACCTGAATCTAAGAAGA-3′ 5′-TGAGAATCCGCTTGTTTTTGAA-3′
Follicular stimulating hormone receptor (*FSHR*)	NM_174061	Forward Reverse	5′-CGACTCTGTCACTGCTCTAACGG-3′ 5′-CGTCAATTCCTTTGGCATAGGTGG-3′
Luteinizing hormone/ choriogonadotropin receptor (*LHCGR*)	NM_174381	Forward Reverse	5′-CAGTCCCCCGCTTTCTCAT-3′ 5′-GTAGAGCCCCATGCAGAAGTCT-3′
Steroidogenic acute regulatory protein (*STAR*)	XR_083945	Forward Reverse	5′-GGATTAACCAGGTTCGGCG-3′ 5′-CTCTCCTTCTTCCAGCCCTC-3′
Cytochrome P450 family 11 subfamily A member 1 (*CYP11A1*)	NM_176644	Forward Reverse	5′-GCCACATCGAGAACTTCCAGAAG-3′ 5′-CTGGTGTGGAACATCTTGTAGACG-3′
Hydroxyl-delta-5-steroid dehydrogenase 3-beta (*HSD3B*)	NM_174343	Forward Reverse	5′-TGTTGGTGGAGGAGAAGGATCTG-3′ 5′-TGGGTACCTTTCACATTGACGTTC-3′
Hydroxysteroid 17-beta dehydrogenase (*HSD17B*)	NM_001102365	Forward Reverse	5′-TTGTGCGAGAGTCTGGCGATTCT-3′ 5′-AGGAATCGCTCGGTGGTGAAGTA-3′
Cytochrome P450 family 17 subfamily A member 1 (*CYP17A1*)	NM_174304	Forward Reverse	5′-TGTGGCCCCTACGCTGAT-3′ 5′-CGCCAATGCTGGAGTCAAT-3′
Cytochrome P450 family 19 subfamily A member 1 (*CYP19A1*)	NM_174305	Forward Reverse	5′-GTCCGAAGTTGTGCCTATTGCCAGC-3′ 5′- CCTCCAGCCTGTCCAGATGCTTGG−3′
Estrogen receptor 1 (*ESR1*)	NM_001001443	Forward Reverse	5'-AGGGAAGCTCCTATTTGCTCC-3' 5'-CGGTGGATGTGGTCCTTCTCT-3'
Fibroblast growth factor 2 (*FGF2*)	NM_174056	Forward Reverse	5′-GAACGGGGGCTTCTTCCT-3′ 5′-CCCAGTTCGTTTCAGTGCC-3′
Prostaglandin E synthase (*PGES*)	NM_174443	Forward Reverse	5′-AGGACGCTCAGAGACATGGA-3′ 5′-TTCGGTCCGAGGAAAGAGTA-3′
Vascular endothelial growth factor A isoform 121 (*VEGFA121*)	NM_174216	Forward Reverse	5′- CCGTCCCATTGAGACCCTG-3′ 5′-CGGCTTGTCACAATTTTTCTTGTC-3′

Follicle wall cell lysis and RNA extraction were conducted according to the manufacturer's recommendations (PureLink RNA Mini Kit, Invitrogen, Carlsbad, CA, USA). Isolated RNA was evaluated for concentration and purity using a NanoDrop One Spectrophotometer (Thermo Fisher Scientific, Waltham, MA, USA). A maximum of 2 μg of mRNA was used to synthesize complementary DNA using a commercial kit (High-capacity cDNA Reverse Transcription Kit, Applied Biosystems, Foster City, CA, USA) supplemented with RNase inhibitor (RNase Inhibitor, human placenta, New England BioLabs, Ipswich, MA, USA). Complementary DNA was used for quantitative real-time reverse transcription PCR using a 7500 Real-Time PCR Detection System (Applied Biosciences) with PowerUp™ SYBR™ Green Master Mix (2X; Applied Biosciences). All assays were carried out in triplicate for each target mRNA. The amplification conditions were as follows: 50°C for 2 min, 95°C for 2 min, and 40 cycles at 95°C for 15 s and 60°C for 60 s.

### Statistical Analyses

Data are presented as percentage mean ± SEM. All statistical analyses were performed using R Version 3.4.3 (https://www.r-project.org/). Normality was confirmed using a Shapiro-Wilk test of the residuals. Non-normal data were transformed using Tukey's Ladder of Powers. If transformation did not result in a normalized population, a Kruskal-Wallis rank-sum test was performed. Analysis of variance was applied to parametric data using a general linear mixed model with repeated measures applied for hormone data. Heifer ID was included as a random variable with each follicle wall piece serving as a replicate for each heifer. Significance was declared at *P* ≤ 0.05.

## Results

**Figure 1:**
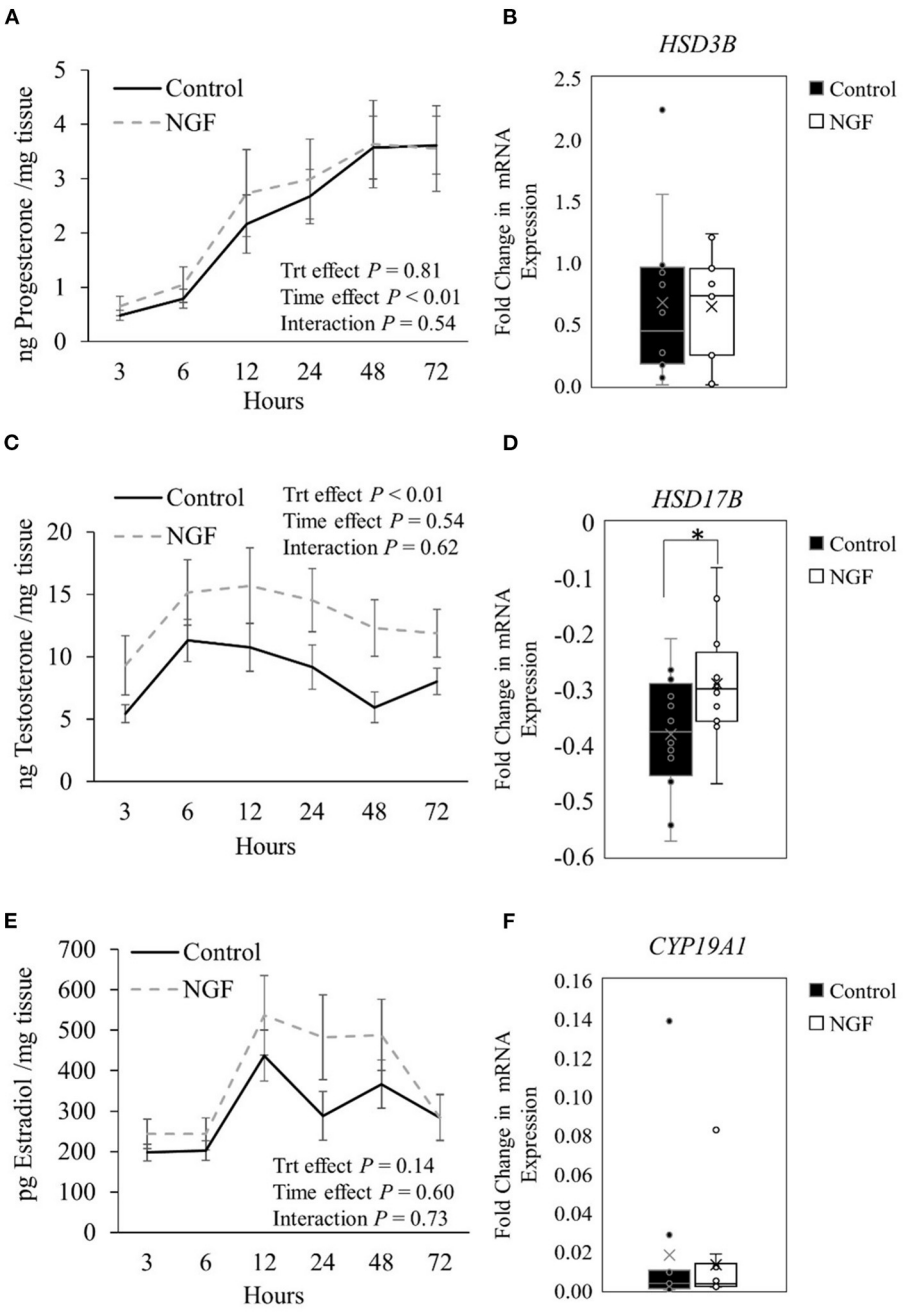
Steroid hormone production **(A,C,E)** and steroidogenic enzyme gene expression **(B,D,F)** in follicle wall cells untreated (control) or treated with 100 ng/mL NGF for 72 h. Hormones are presented as mean ± SEM, whereas genes are presented as box-and-whisker plots. *P ≤ 0.05.

Progesterone production from follicular wall cells (Figure 1A) changed over time in the culture system (*P* < 0.01) but was not altered by NGF treatment (*P* = 0.81) or treatment by time interactions (*P* = 0.54). Consistently, there were no changes in gene expression of steroidogenic enzymes responsible for the conversion of cholesterol to pregnenolone *(STAR, CYP11A1*; *P* ≥ 0.34; Table 2*)* or pregnenolone to progesterone *(HSD3B; P* = 0.60, Figure 1B) after treatment with NGF for 72 h. Testosterone production in NGF-treated follicular wall cells (Figure 1C) was higher than in untreated controls (*P* < 0.01), but not affected by time (*P* = 0.54) or treatment by time interactions (*P* = 0.62). While there was no change in follicular *CYP17A1* expression (*P* = 0.31; Table 2), whose enzyme converts progesterone to androstenedione, NGF treatment upregulated follicular *HSD17B* expression (*P* = 0.04; Figure 1D), whose enzyme converts androstenedione to testosterone in the theca cells (13). Follicular estradiol production (Figure 1E) was unaffected by NGF treatment (*P* = 0.14), time (*P* = 0.60), or treatment by time interactions (*P* = 0.73). Consistently, follicular *CYP19A1* expression was also unaffected by NGF treatment (*P* = 0.53; Figure 1F).

**Table 2:** Fold change in mRNA expression of gonadotropin receptors, angiogenic enzymes, and steroidogenic enzymes in bovine follicle wall cells preparations treated with 100 ng/mL NGF vs. untreated (Control) for 72 h.

**Gene names and abbreviations**	**Control**	**NGF**	***P*-value**
Steroidogenic acute regulatory protein (*STAR*)	0.17 ± 0.02	0.29 ± 0.1	0.34
Cytochrome P450 family 11 subfamily A member 1 (*CYP11A1*)	17.81 ± 9.2	8.67 ± 5.0	0.40
Cytochrome P450 family 17 subfamily A member 1 (*CYP17A1*)	0.01 ± 0.00	0.01 ± 0.00	0.31
Luteinizing hormone/ choriogonadotropin receptor (*LHCGR*)	0.07 ± 0.01	0.09 ± 0.03	0.41
Follicular stimulating hormone receptor (*FSHR*)	Undetected	Undetected	N/A
Estrogen receptor alpha (*ESR1*)	0.04 ± 0.01	0.05 ± 0.02	0.77

*Data is presented as mean ± SEM*.

Follicular expression of *LHCGR* was unaltered by treatment after 72 h culture (*P* = 0.41), whereas *FSHR* was undetectable by the assay (Table 2). We also found no increase in the expression of *ESR1* following the 72-h culture period in either treatment group (*P* = 0.77; Table 2).

**Figure 2:**
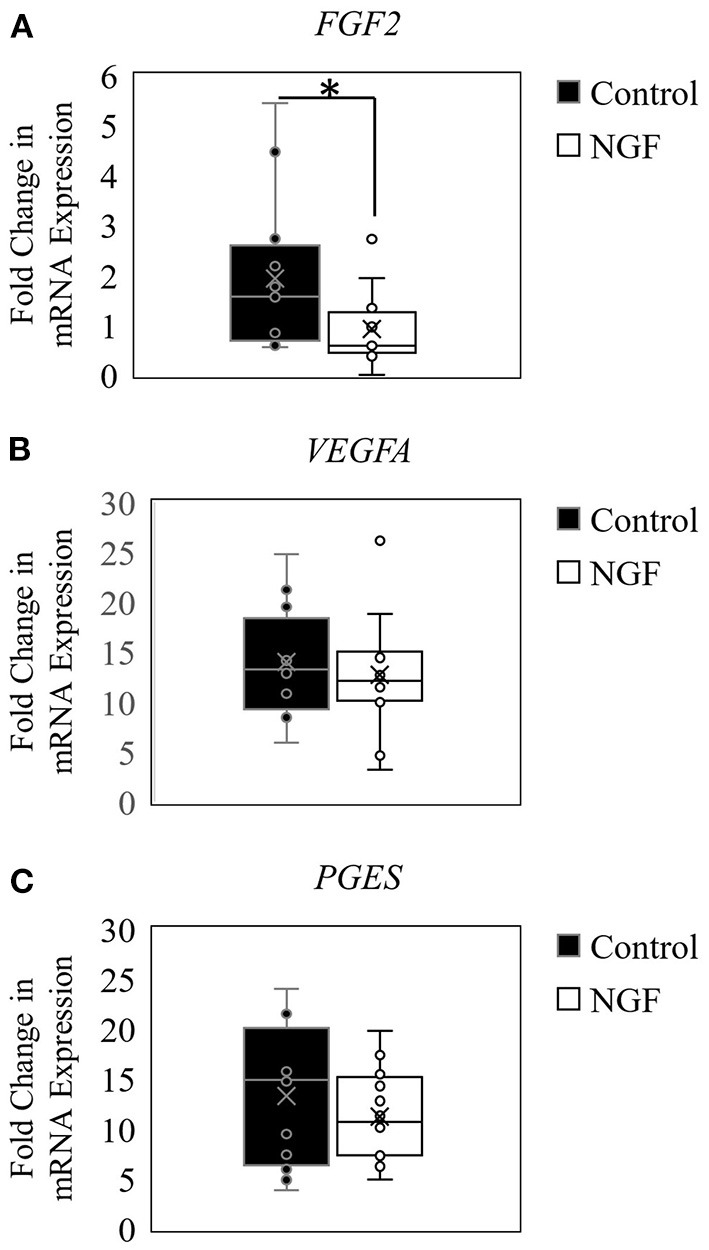
Box-and-whisker plots demonstrating gene expression of angiogenic enzymes FGF2 **(A)**, VEGFA **(B)**, and PGES **(C)** in follicle wall cells untreated (control) or treated with 100 ng/mL NGF for 72 h. *P ≤ 0.05.

The results herein also demonstrated that NGF treatment down-regulated expression of *FGF2* (*P* = 0.02; Figure 2A), but did not alter *VEGFA121* (*P* = 0.44; Figure 2B) or *PGES* (*P* = 0.63; Figure 2C) expression in bovine follicle wall cells.

## Discussion

The current study evaluated the direct effects of NGF, purified from bull seminal plasma, on bovine pre-ovulatory follicle wall cells. In a previous study, androstenedione and progesterone production was increased in isolated bovine theca cells treated with recombinant NGF (3). Androstenedione is converted to testosterone in the theca cells by the enzyme hydroxysteroid 17-beta dehydrogenase (HSD17B) (13), both of which were increased in the current study. Testosterone produced by theca cells is typically converted to estradiol in the granulosa cell via the aromatase enzyme (13). Despite the increased production of its testosterone precursor, estradiol concentrations, and aromatase enzyme gene expression (*CYP19A1*) were unaffected by NGF treatment. Previously, *ESR1* expression was observed in the theca interna cells of growing pre-ovulatory follicles in the bovine ovary and thought to provide a local feedback loop, whereby estradiol produced by granulosa cells could be used to further stimulate theca cell androgen production (16). In that study, highest expression of *ESR1* was detected during follicular growth and in the early luteal phase (day 1 through 4 of the estrous cycle) (16). The current study did not find an effect of NGF treatment of *ESR1* expression in bovine follicular wall cells, further suggesting that this feedback loop is not the potential signaling mechanism for NGF-induced androgen production. However, NGF stimulated proliferation of theca cells from bovine pre-ovulatory follicles (3). Therefore, the increase in testosterone production observed herein could be due to an increase in theca cell number, since estradiol production by the granulosa cells was unaffected.

Treatment of follicle wall cells with NGF did not alter the expression of other steroidogenic enzymes or progesterone production, which serves as a precursor for androstenedione production in the preovulatory follicle (13). This is contradictory to the previous observation of increased theca cell progesterone production in response to NGF treatment (3). One difference between studies is that our culture media contained gonadotropins (LH/FSH) in both control and NGF wells, attempting to emulate the pre-ovulatory cascade, whereas the previous study treated only the control wells with gonadotropins (3). Supplementing culture media with LH and FSH stimulated endogenous NGF secretion from medium to large follicles in ewes (5). Therefore, the inclusion of gonadotropins in the control medium, but not in the NGF-treated medium, may confound the outcomes of exogenous NGF supplementation since the ovary is capable of endogenous NGF production in response to gonadotropin signaling. It is also worth noting that human chorionic gonadotropin (hCG) was used to treat control samples previously rather than LH (3). While hCG shares the same receptor with LH, hCG also stimulates different intracellular signaling pathways (17), which could alter its downstream effects. Lastly, the current study utilized purified, rather than recombinant NGF to assess species-specific functions of this protein in bovine reproduction. The purified form used in the current study was previously found to be ~59% pure (7). The major contaminants were found to be a c-type natriuretic peptide (16.41%) and a serine protease inhibitor (11.26%).

Interestingly, the c-type natriuretic peptide has been found to inhibit oocyte meiotic resumption in mice (18) and pigs (19), but stimulate oocyte meiotic resumption in cattle (20). Serine protease inhibitors were likely a contaminant from protease inhibitors used during seminal plasma harvest and can have differing effects on fertilization rates when supplemented *in vitro* (21). While we speculate that the NGF is the primary component causing the results observed in the current study, there is still a need to further elucidate the function of each seminal plasma component through recombinant protein development.

In cattle, ovarian function depends on a complex remodeling of the vascular system between ovulation and CL development that involves the temporal expression of vascular endothelial growth factor A (VEGFA) and fibroblast growth factor 2 (FGF2) (22). Interestingly, there was a downregulation in gene expression of *FGF2* and no change in gene expression of *VEGFA121* in follicle wall cells treated with NGF. Both VEGFA and FGF2 promote vascular supply growth during follicular to luteal transition in the cow ovary, with resulting changes in their localization patterns (23, 24). Follicular *FGF2* mRNA and FGF2 protein increased around 4 h after GnRH administration in cows, corresponding with the LH surge (25). Immediately following the LH surge, FGF2 stimulates the migration and proliferation of endothelial cells that help to establish luteal blood flow (22). During early CL formation, FGF2 concentrations decrease while the capillary beds are reconstructed to establish blood flow (22). In contrast, VEGFA concentrations remain high throughout ovulation and CL development to support endothelial cell survival (22). Given that the assays were performed after 72 h in culture, it is possible that the timing of this reconstruction phase was hastened by NGF treatment, which may account for the observed decrease in *FGF2* expression. Future studies assessing the temporal expression of these angiogenic enzymes are warranted to clarify exactly how NGF could alter the follicular to luteal transition.

Another factor crucial to ovulation and CL formation is PGE, which can be stimulated from bovine theca cells with NGF treatment (3, 26). Previously, NGF treatment stimulated PGE release for up to 8 h in theca cells extracted from bovine preovulatory follicles (3). Prostaglandin E_2_ is synthesized by PGES and acts as a pro-angiogenic molecule in vascular endothelium by recruiting the paracrine-autocrine mechanism characteristic of endothelium cells, resulting in vascular remodeling (27). Prostaglandin E_2_ also supports luteal progesterone production in cattle (28), potentially through increased CL vascularity (29). Consistently, one study observed higher *PGES* mRNA and PGES protein levels in the CL of early pregnancy (days 20–30) than in the luteal phase (days 8–12 of the estrous cycle) or after 40 days gestation of artificially inseminated cows (30). To our surprise, NGF treatment did not enhance follicular expression of *PGES* in the current study. However, HSD17B enzymes have also been found to play a role in the synthesis of arachidonic acid and its downstream eicosanoid metabolites, such as PGE (31). Additionally, HSD17B-knockout female mice failed to initiate pseudopregnancy after being mated by sterile males despite exhibiting normal cycles (32), suggesting a crucial role of this enzyme in CL development. Therefore, NGF may influence ovulation and CL development indirectly through its effects on ovarian HSD17B enzyme activity.

The cultured follicle wall was derived from follicles destined to ovulate within 24 h based on the synchronization protocol used. Therefore, by 72 h, we expected to see changes consistent with the post-ovulatory follicular to luteal transition. Following ovulation, the LH-receptor-bearing theca cells luteinize and become small luteal cells (33, 34). On the other hand, FSH-receptor-bearing granulosa cells luteinize and become large luteal cells, losing their FSH receptors (35). Small luteal cells, in response to LH binding, produce an early rise in progesterone that is essential for supporting initial embryonic growth (36). Though we found no differences in expression of *LHCGR* in the current study, it would be worthwhile to evaluate its expression *in vivo* to determine if there are downstream effects on the presence of small luteal cells in the mature bovine CL. This finding may explain how systemic administration of NGF can improve CL development and function in cattle (6, 7, 37).

In conclusion, the results of the current study demonstrated that purified bovine NGF could act directly on the theca and granulosa cells of the bovine pre-ovulatory follicle to stimulate testosterone production, which may be secondary to theca cell proliferation. Additionally, decreased *FGF2* expression in NGF-treated follicle wall cells suggests hastened onset of follicle wall cellular remodeling that occurs during early luteal development.

## Data Availability Statement

The original contributions presented in the study are included in the article/supplementary materials, further inquiries can be directed to the corresponding author.

## Ethics Statement

The animal study was reviewed and approved by Institutional Animal Care Use Committee.

## Author Contributions

JS, FL, and IC conceptualize the study. All authors contributed to the article and approved the submitted version.

## Funding

This work was supported by the National Institute of Food and Agriculture at the U.S. Department of Agriculture Hatch Funds (Accession number: 1014712).

## Conflict of Interest

The authors declare that the research was conducted in the absence of any commercial or financial relationships that could be construed as a potential conflict of interest.

## Publisher's Note

All claims expressed in this article are solely those of the authors and do not necessarily represent those of their affiliated organizations, or those of the publisher, the editors and the reviewers. Any product that may be evaluated in this article, or claim that may be made by its manufacturer, is not guaranteed or endorsed by the publisher.
